# The effects of beliefs about AIDS-related death on quality of life in Chinese married couples with both husband and wife infected with HIV: examining congruence using the actor-partner interdependence model

**DOI:** 10.1186/s12955-017-0703-z

**Published:** 2017-06-17

**Authors:** Nancy Xiaonan Yu

**Affiliations:** Department of Applied Social Sciences, City University of Hong Kong, Tat Chee Avenue, Kowloon, HKSAR China

**Keywords:** Beliefs about AIDS-related death, Quality of life, Mental health, Married couple, Actor-partner interdependence model

## Abstract

**Background:**

This cross-sectional study examined the actor and partner effects of beliefs about AIDS-related death on quality of life in Chinese married couples in which both were living with HIV.

**Methods:**

A total of 49 married couples in central China who were both infected with HIV completed measures to assess their beliefs about AIDS-related death and quality of life.

**Results:**

In the actor-partner interdependence model, the husband-wife dyad showed congruence in their beliefs about AIDS-related death (*r* = .40) and quality of life–mental health summary (*r* = .31), respectively, within the couple. Both actor and partner effects of beliefs about AIDS-related death on the quality of life–mental health summary, rather than the quality of life–physical health summary, were significant within the husband-wife dyad.

**Conclusions:**

Our findings indicate the dyadic interdependence of beliefs about AIDS-related death and the quality of life–mental health summary in married couples. Psychosocial interventions that target a reduction of negative death beliefs and enhancement of well-being in the context of HIV should treat the couple as a unit.

## Background

Due to pessimism about the prognosis of HIV and the wide stigma attached to this disease, people with HIV tend to present negative beliefs about death. This is particularly significant among people who were accidentally infected with HIV while giving a commercial blood donation to ease their financial strain [1] and are now living with scarce medical and psychosocial care in rural China. In Chinese culture, which values family orientation, congruence within couples who experience major stressors has not been examined empirically. For Chinese married couples who are both infected with HIV and cope with HIV as a unit, the husband-wife dyad may have similar beliefs about death and show congruent quality of life (QoL). Guided by the family systems theory [2], this study aimed to examine the dyadic interdependence of beliefs about AIDS-related death and QoL in Chinese couples who both have HIV.

Living with HIV can be torturous, and dying of AIDS may also be attached with tremendous suffering. Dying AIDS patients have been described in extremely negative ways, such as, “When they get sick it’s really scary. Their skin and face become white and start to peel off layer by layer,” and “She couldn’t eat anything. She became so thin that it was frightening” [3]. Misunderstanding and panic might lead to beliefs about unpleasant AIDS-related death compared to other illnesses such as cancer. A patient infected with HIV might rather choose cancer and die within a month, because the shame from the “dirty disease” of HIV/AIDS is difficult to bear [4]. Denial, fear, and fatalism of AIDS-related death have been widely documented in many cultures; for example, people with HIV have been depicted as “dead before dying” in South Africa [5]. Negative beliefs about AIDS-related death might undermine the QoL of people with HIV who are confronted with the threat of approaching death, which has not been reported in the HIV literature.

Husband and wife are both affected by chronic illness because the disease has a wide impact on a couple [6]. The family systems theory posits that chronic illness affects not only the individual but the whole family [2]. The interdependence between cancer patients and caregivers, for instance, on illness representation, coping, and mental health, has received attention in health psychology [7–9]. The stigmatized nature of HIV stains the entire family after an individual receives a diagnosis of HIV infection [10], particularly in the Chinese culture, in which family defines the central identity. This is plausible in couples in which both partners are infected with HIV and thus carry the dual role of patient and caregiver. Such a couple may form a closer bond to face the various challenges related to the disease. The congruence between couples who are both infected with HIV is understudied [11].

**Fig. 1:**
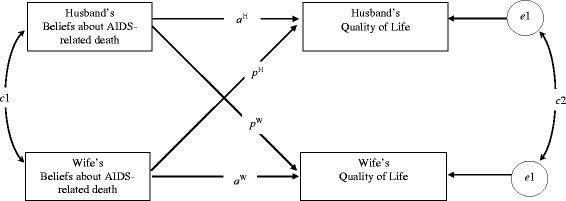
Hypothesized model of dyadic effects of beliefs about AIDS-related death and quality of life in married couple with both the husband and wife infected with HIV using the actor-partner interdependence model. *a* ^H^: Actor effect of the husband’s beliefs about AIDS-related death on his own quality of life. *a* ^W^: Actor effect of the wife’s beliefs about AIDS-related death on her own quality of life. *p* ^H^: Partner effect of the wife’s beliefs about AIDS-related death on her husband’s quality of life. *p* ^W^: Partner effect of the husband’s beliefs about AIDS-related death on his wife’s quality of life

The actor-partner interdependence model (APIM) [12] has been widely used to investigate the bidirectional effects in dyadic data. The APIM treats the dyad rather than the individual as the unit of analysis. In APIM analysis, the two members of a pair are regarded statistically relative to each other within the dyad. The “actor effect” is defined as the effect of a person’s own variables on his or her own outcome, that is, the effect within the individual. In contrast, the “partner effect” refers to the effect of the partner’s variable on a person’s outcome, indicating the effect across individuals within the dyad. For this study, in which the married couple is treated as a dyad, the husband’s beliefs about death, for example, may influence not only his own QoL (actor effect [13]), but also his wife’s QoL (partner effect [6]), and vice versa. Specifically, the actor effects tested are: 1) the husband’s beliefs about AIDS-related death on his own QoL (*a* ^H^) and 2) the wife’s beliefs about AIDS-related death on her own QoL (*a* ^W^). In addition, the partner effects examined are: 1) the husband’s beliefs about death on his wife’s QoL (*p* ^W^), and 2) the wife’s beliefs about death on her husband’s QoL (*p* ^H^) (Fig. 1).

By examining dyadic interdependence in a sample of Chinese married couples who are both infected with HIV, we hypothesized that 1) the husband-wife dyad will show significant congruence in their beliefs about AIDS-related death and QoL, respectively; and 2) significant actor and partner effects of beliefs about AIDS-related death on QoL will be seen within the husband-wife dyad (Fig. 1).

## Methods

A total of 49 couples who were both infected with HIV completed this cross-sectional survey. The study was carried out in seven villages in Henan Province, central China. These villages were randomly selected from 22 villages that had an HIV prevalence greater than 10%. These people were infected with HIV when one spouse got infected while giving a commercial blood donation and then transmitted HIV to the other spouse [1]. The inclusion criteria were 1) HIV infection in both husband and wife, and 2) married status for more than 5 years. The couples were randomly selected from the registry of patients with HIV maintained in the village offices. The response rate of the married couples was 71% (49 of 69 couples). The couples provided written informed consent to participate in this study. The spouses were interviewed separately. The participants received compensation for their time spent in this survey (CNY30, approximately equivalent to US$5) Ethics approval was obtained from City University of Hong Kong.

### Main outcome measures

***Negative beliefs about AIDS-related death*** was measured using five items that had been used in a sample of Chinese AIDS-bereaved family members [14]. After they were asked to compare AIDS-related death with that of other illnesses such as cancer and coronary heart disease, the participants indicated their level of agreement (1 = do not agree at all, 5 = agree very much) on five items, “AIDs patients die earlier,” “AIDS patients die with more pain,” “AIDS patients die with less dignity,” “Dying AIDS patients are more miserable,” and “AIDS deceased have shabbier funerals.” The sum score was used to represent negative beliefs about death, with higher scores indicating more negative beliefs. Cronbach’s α value was .88 in husbands and .86 in wives, showing good internal consistency of these measures [15].

***QoL*** was measured using the 35-item Medical Outcomes Study HIV Health Survey [16]. The Chinese version showed good psychometric properties [17]. The composites for the QoL–physical health summary and the QoL–mental health summary were computed, and higher scores indicate higher levels of physical health or mental health. The subscales in this study had Cronbach’s α values greater than .70 in both husbands and their wives, showing acceptable internal consistency [15].

### Statistical analysis

The couples’ demographic and HIV-related characteristics were tabulated. The Pearson correlations were used to test for bivariate associations between beliefs about AIDS-related death and the two dimensions of the QoL measure among the couples. The APIM approach [18] was applied to test the dyadic effects of these beliefs on QoL within the married couple. We estimated the actor effects and partner effects of beliefs about AIDS-related death on QoL within the husband-wife dyad. These models controlled for demographic (i.e., age, education, family income, duration of marriage) and HIV-related characteristics (i.e., duration since HIV diagnosis). The model fit was evaluated using several fit indices: 1) the chi-square (χ^2^) test of model fit, 2) the root mean square error of approximation (RMSEA), 3) the comparative fit index (CFI), 4) the Tucker-Lewis index (TLI), and 5) the standardized root mean square residual (SRMR). A good fit is demonstrated with an RMSEA value of less than .06, CFI of .95 or above, TLI of .90 or higher, and SRMR value below .08 [19]. We conducted data analyses using Mplus 7; *p* values of less than .05 were considered to indicate statistical significance. According to Cohen’s guidelines to interpret effect size [20], .10 is small, .30 is medium, and .50 is large.

## Results

Table 1 presents the demographics and HIV-related information of the couples. Most were middle-aged, had a low level of education, and reported a low family income. They had been infected with HIV more than 13 years earlier. The duration of their marriage was more than 23 years (Table 1).

**Table 1:** Demographic characteristics and HIV-related information of the husbands and their wives (49 of each)

	Husbands	Wives
	*n* (%) or Mean ± SD	*n* (%) or Mean ± SD
Age (years)	45.78 ± 7.56	45.08 ± 7.51
Education		
Elementary or lower	21 (42.9%)	27 (55.1%)
Secondary or higher	28 (57.1%)	22 (44.9%)
Duration since HIV diagnosis (years)	13.94 ± 2.14	13.49 ± 1.98
Family income (RMB)	2051.02 ± 631.18	–
Duration of marriage (years)	23.04 ± 7.73	–

1US$ = 6.1RMB

In Table 2, the husband-wife dyad showed significant correlation in their beliefs about AIDS-related death (*r* = .40, *p* < .01) and their QoL–mental health summary (*r* = .51, *p* < .01), but not in their QoL–physical health summary (*r* = .16, *p* > .05). Although not significantly associated with the QoL–physical health summary (*r* = −.12, *p* > .05), the husbands’ beliefs about AIDS-related death was negatively associated with their QoL–mental health summary (*r* = −.39, *p* < .01). The wives showed a similar pattern in which their beliefs about AIDS-related death were not significantly associated with their QoL–physical health summary (*r* = −.26, *p* > .05), but were negatively associated with their QoL–mental health summary (*r* = −.50, *p* < .01). Therefore, we excluded the results of the QoL–physical health summary from further analysis with the APIM.

**Table 2:** Descriptive statistics and bivariate correlations of study variables of husbands (H) and their wives (W)

	Mean ± SD	1	2	3	4	5
1. Beliefs about AIDS-related death (H)	9.55 ± 4.50	–				
2. Quality of life-physical health summary (H)	39.77 ± 10.61	−.12	–			
3. Quality of life-mental health summary (H)	46.16 ± 9.90	−.39**	.61***	–		
4. Beliefs about AIDS-related death (W)	9.48 ± 3.82	.40**	−.14	−.47***	–	
5. Quality of life-physical health summary (W)	37.51 ± 8.90	−.25	.16	.26	−.26	–
6. Quality of life-mental health summary (W)	47.40 ± 10.30	−.44**	.07	.51***	−.50***	.52***

**, *p* < .01; ***, *p* < .001

**Fig. 2:**
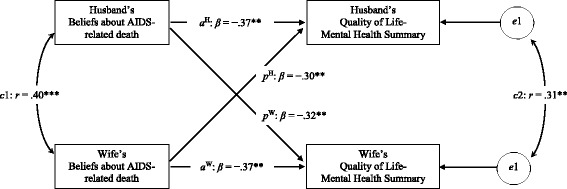
Beliefs about AIDS-related death and quality of life-mental health summary: Congruence in the husband-wife dyad using the actor-partner interdependence model Paths labelled *a* indicate the actor effects and paths labelled *p* indicate partner effects for husband (H) and wife (W), and standardized regression coefficients (beta) are shown. Path *c*1 represents the association between the husband’s and wife’s beliefs about AIDS-related death, and the correlation coefficient is indicated. Path *c*2 represents the association between residual errors of the husband’s and wife’s quality of life-mental health summary, and the correlation coefficient of the residual errors is indicated. **, *p* < .01; ***, *p* < .001

In APIM analysis using Mplus 7, the model fit the data well (χ^2^ [1] = 1.09, *p* = .30; RMSEA = .04; CFI = .99; TLI = .98; SRMR = .06) [19]. All actor and partner effects of beliefs about AIDS-related death on the QoL–mental health summary are significant (Fig. 2), indicating that individuals’ beliefs about AIDS-related death were not only associated with a low score on their own QoL–mental health summary, but also with a low score on that of their spouses. The results remained identical after controlling for covariates of demographic and HIV-related characteristics of the husband and wife.

## Discussion

Focusing on a dyadic perspective, this study provides new information about the interdependence of Chinese married couples who are both infected with HIV. The results show that 1) both members of the husband-wife dyad reported a moderate level of congruence in their beliefs about AIDS-related death and their score on the QoL–mental health summary, respectively; and 2) the husbands’ beliefs about AIDS-related death affected not only their own QoL–mental health summary but also those of their wives, and vice versa.

As expected, the couples shared beliefs about AIDS-related death, indicating that they present a similar cognitive scheme regarding the HIV prognosis and finality. In addition, the couples showed congruent QoL, particularly in the mental health summary, which is consistent with the results of previous dyadic studies on chronic illness [21, 22]. This result reveals that the married couple is yoked to experience emotional contagion in the process of adjusting to the disease [23]. This is particularly true in the Chinese culture, which values the wholeness of a couple, especially when they experience difficulties together.

Notably, significant actor and partner effects were shown in the effects of beliefs about AIDS-related death on the QoL–mental health summary in the married couples. Because both husbands and their wives were infected with HIV, the actor and partner effects are comparable in strength, unlike the dyad of patients and caregivers, which shows higher actor effects than partner effects [24]. These new findings provide support for the family systems theory that the couples develop interdependence in the face of family stress [2]. Interaction within the couple is often overlooked in existing HIV management and service [25]. Our findings indicate the need to treat the married couple as a unit in psychosocial intervention programs to provide better HIV care. Future studies could consider the use of a qualitative approach to understand more about the dynamics within the husband-wife dyad and the mechanism of the effects of illness-related conceptualization on their own and their partner’s quality of life and well-being outcomes.

This study used a small convenience sample, which is not representative of the couples infected with HIV. We did not calculate the statistical power before this pilot study because couples with both members infected with HIV were difficult to recruit. In addition, the moderate response rate suggests that there may be a bias in recruitment and that the results should thus be interpreted with caution. The cross-sectional design of this study does not inform directions of effects; for example, a couple’s poor quality of life might drive their negative beliefs about death. Longitudinal studies are needed to examine the prospective effects of the key variables in the husband-wife dyad. In addition, this study relied on self-reporting, and future studies may consider the use of physiological or other objective indicators to observe the dyadic interdependence.

## Conclusions

Despite these limitations, by indicating the actor and partner effects of beliefs about AIDS-related death on QoL within married couples who are both infected with HIV, this study contributes to the growing knowledge about dyadic interdependence in the context of HIV. The mutual influences in the dyadic relationships affected by HIV require further investigation. In addition, these findings have public health implications in the design of couple-based intervention programs for HIV prevention and treatment.

## Abbreviations

APIM: Actor-partner interdependence model
CFI: Comparative fit index
QoL: Quality of life
RMSEA: Root mean square error of approximation
TLI: Tucker-Lewis index
SRMR: Standardized root mean square residual
